# Investigation of GaInAs strain reducing layer combined with InAs quantum dots embedded in Ga(In)As subcell of triple junction GaInP/Ga(In)As/Ge solar cell

**DOI:** 10.1186/s11671-015-0821-7

**Published:** 2015-03-07

**Authors:** Senlin Li, Jingfeng Bi, Mingyang Li, Meijia Yang, Minghui Song, Guanzhou Liu, Weiping Xiong, Yang Li, Yanyan Fang, Changqing Chen, Guijiang Lin, Wenjun Chen, Chaoyu Wu, Duxiang Wang

**Affiliations:** Xiamen San’an Optoelectronics Co., Ltd, Xiamen, 361009 China; Tianjin San’an Optoelectronics Co., Ltd, Tianjin, 300384 China; Wuhan National Laboratory for Optoelectronics, School of Optical and Electronic Information, Huazhong University of Science and Technology, Wuhan, 430074 China

**Keywords:** InAs quantum dots, Triple junction solar cell, Strain reducing layer, Metal organic chemical vapor deposition

## Abstract

The InAs/GaAs quantum dots structure embedded in GaInP/Ga(In)As/Ge triple junction solar cell with and without Ga_0.90_In_0.10_As strain reducing layer was investigated. Conversion efficiency of 33.91% at 1,000 suns AM 1.5D with Ga_0.90_In_0.10_As strain reducing layer was demonstrated. A 1.19% improvement of the conversion efficiency was obtained via inserting the Ga_0.90_In_0.10_As strain reducing layer. The main contribution of this improvement was from the increase of the short-circuit current, which is caused by the reduction of the Shockley–Read–Hall recombination centers. Consequently, there was a decrease in open circuit voltage due to the lower thermal activation energy of confined carriers in Ga_0.9_In_0.1_As than GaAs and a reduction in the effective band gap of quantum dots.

## Background

Today’s state-of-the-art conversion efficiency of matured lattice-matched GaInP/Ga(In)As/Ge solar cell is 41.6% ± 2.5% at 364 suns concentration and 32.0% ± 1.5% at one sun [1], which have been widely used for terrestrial photovoltaic [2] and space applications [3]. Experimentally, the outdoor module performance had strong relations with the Ga(In)As subcell in CPV power plants. In this case, for further efficiency enhancement, quantum structures such as quantum well [4] and quantum dots (QDs) [5-14] are usually introduced to extend the absorption spectrum to lower energy photons, thus increasing the current density. Additionally, the QDs enhanced devices may improve the radiation tolerance for space application, temperature coefficients, and spectral response under concentration [14-16]. However, the incorporation of quantum structures is usually accompanied by a significant decrease in open-circuit voltage (V_oc_). For these reasons, numerous efforts have been made inducing a high-potential barriers fence [5-7] and strain compensation technique [9-11]. As reported, the V_oc_ could recover via the decrease in trapping and recombination of carriers in the QDs and wetting layer, which is due to resonant tunneling through a new electronic state created by the fence layer. For the strain compensation technique, the V_oc_ might maintain by the decrease of Shockley-Read-Hall (SRH) recombination that benefit from the less defects. Almost all these mentioned above quantum dots solar cell are based on a GaAs substrate. When InAs/GaAs QD structure is grown on a Ge substrate, GaAs become the natural compensation materials for its lattice constant between InAs and Ge. Nevertheless, the larger lattice mismatch between InAs QDs and GaAs space layer usually induces defects. These defects become the trap center for photon generated carries, thus deteriorating the performance of device. In this paper, a Ga_0.90_In_0.10_As strain reducing layer (SRL) was applied to reduce these defects in the QD solar cell. The effects of Ga_0.90_In_0.10_As SRL on InAs QDs embedded in GaInP/Ga(In)As/Ge triple junction solar cell (TJSC) were investigated.

## Methods

**Figure 1:**
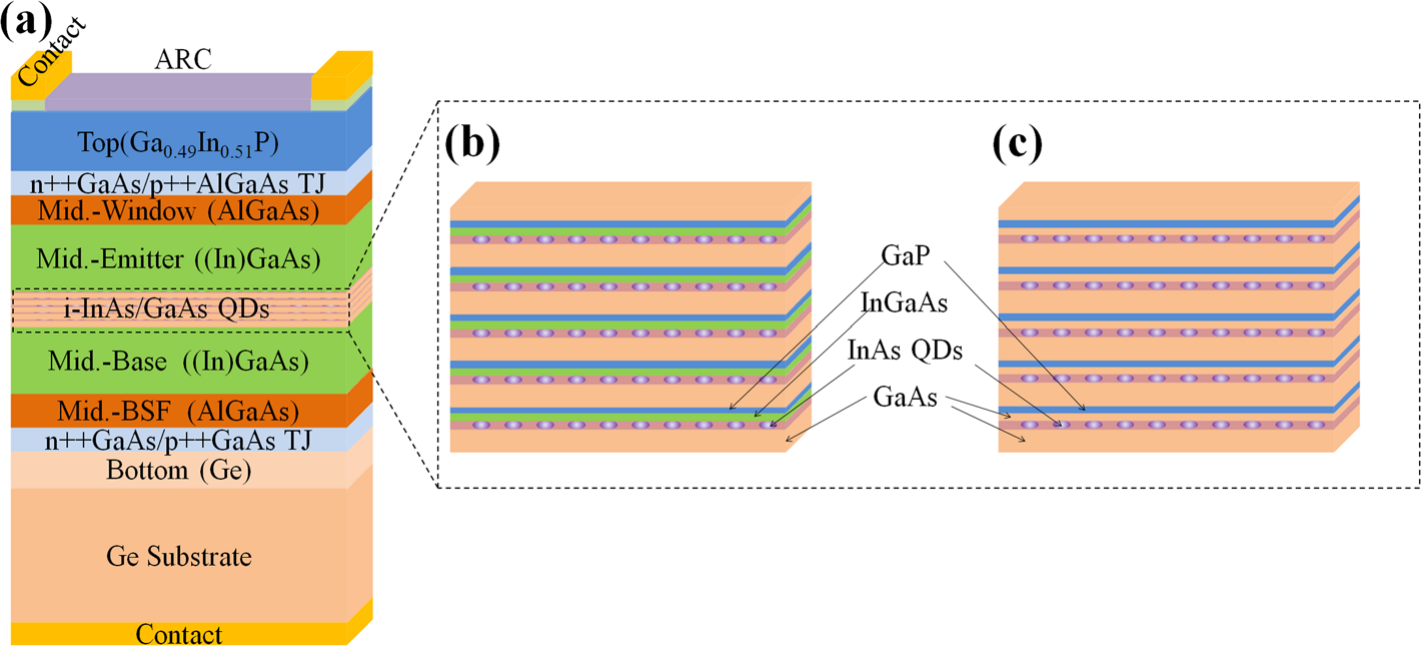
**The QD TJSC image. (a)** The structure of QD TJSC, QD structure of **(b)** sample A1 with Ga_0.90_In_0.10_As SRL, **(c)** sample A2 without Ga_0.90_In_0.10_As SRL.

The QD TJSC was fabricated on a 4 in. Ge (001) substrate 9° offcut toward <111> direction via metal organic chemical vapor deposition (MOCVD). As shown in Figure 1, the Ge subcell and *p-i-n* Ga(In)As subcell are connected by the n++GaAs/p++GaAs tunnel junction (TJ), and the *p-i-n* Ga(In)As subcell and GaInP subcell are connected by the n++GaAs/p++AlGaAs TJ. A five-layer InAs QDs with and without Ga_0.90_In_0.10_As SRL embedded in the *i*-region of *p-i-n* Ga(In)As subcell was studied.

Prior to the InAs QD structure growth, Ge subcell, n++GaAs/p++GaAs TJ, AlGaAs back surface field, and Ga(In)As base were formed on the Ge substrate, and then a 15 nm intrinsic GaAs was deposited at 620°C. Thereafter, the substrate temperature was lowed 510°C for the InAs QDs growth via Stranski-Krastanov growth mode with a coverage of 1.8 monolayer, followed by a 3 nm Ga_0.90_In_0.10_As SRL, an 1 nm CaP strain compensation layer, and an 11 nm GaAs layer, and then the substrate temperature was ramped to 620°C for a high temperature GaAs growth with a thickness of 25 nm. By repeating this sequence, a five periods of InAs QDs were fabricated in the intrinsic region. For the surface and strain analysis, the fifth layer InAs QDs was uncapped, denoted as sample A1. For a comparison, the 3 nm Ga_0.90_In_0.10_As SRL in sample A1 was replaced by a 3 nm GaAs layer with all the other growth parameters remained the same, denoted as sample A2. Then, the Ga(In)As emitter and AlGaAs window were deposited, followed by a n++GaAs/p++AlGaAs TJ, GaInP subcell, and GaAs contact layer, completing the QD TJSC growth. The QD TJSC that based on sample A1 is denoted as sample B1, and that based on sample A2 is denoted as sample B2.

Post MOCVD growth, the structure was processed into chips with size of 1 × 1 cm^2^. The front grids were patterned by photolithography and chemical etch with a shadow ratio of 5%, and then the contact metal was deposited by thermal evaporation. Subsequently, an Al_2_O_3_/Ti_3_O_5_ dual layer anti-reflection coating (ARC) was evaporated on the front surface of the cells forming the front-side passivation of the top cell to improve the spectral response. All the solar cells were isolated by a chemical etch.

The InAs QD densities and morphologies of sample A1 and A2 were characterized by atomic force microscopy (AFM). The optical performance was measured by a room temperature (RT) photoluminescence (PL). The structural characterization like the strain condition was analyzed by high resolution X-ray diffraction (HRXRD). The external quantum efficiency (EQE) of QD TJSC was measured with a commercial Enlitech system. The current-voltage (I-V) characteristics were measured using a solar simulator at RT under 1,000 suns, low-AOD, AM 1.5D conditions.

## Results and discussion

**Figure 2:**
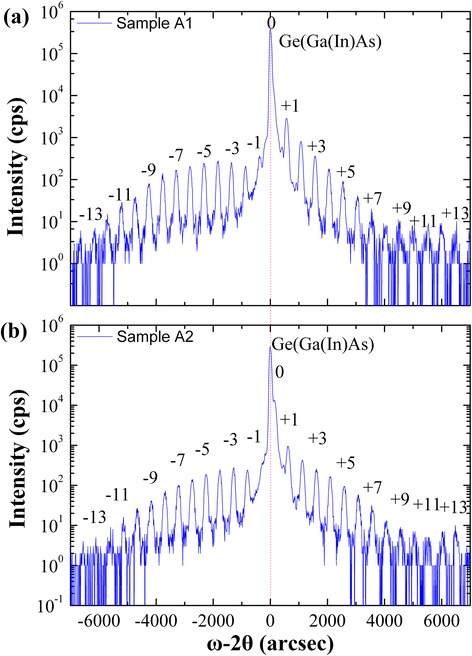
**HRXRD of InAs/GaAs QD structure. (a)** Sample A1 with Ga_0.90_In_0.10_As SRL, **(b)** sample A2 without Ga_0.90_In_0.10_As SRL.

As illustrated in Figure 2, the Ga(In)As and Ge are lattice matched which were confirmed by the single Bragg peak. In both HRXRD spectra, the five period InAs/GaAs QD superlattice (SL) fringe peaks are visible. The zeroth-order peak of sample A1 is buried underneath the Ga(In)As and Ge Bragg peak, but a shift of 140 arc-second is observed in sample A2, meaning that the five period InAs/GaAs QD SL is under a tensile strain from the substrate. The out-of-plane strain *ε*_⊥_ is 1.045‰, which is determined from a differentiated formulation of Bragg’s law shown as followed [17]:

1 $$ {\varepsilon}_{\perp }=\varDelta a/a=\varDelta {\theta}_{sl} cot{\theta}_b $$

Where Δθ_sl_ is the difference in angle between the substrate Bragg peak and the zeroth order SL peak, θ_b_ is the value of the substrate Bragg angle, and Δa/a is the fractional lattice mismatch representing the out-of-plane strain of the SL. Corresponding the in-plane strain *ε*_//_ (1.136‰) of InAs QDs can be estimated by the equation [18]

2 $$ {\varepsilon}_{\perp }=-2\frac{C_{12}}{C_{11}}{\varepsilon}_{//} $$

These results suggested that more defects may be formed in the QD TJSC without Ga_0.90_In_0.10_As SRL.

**Figure 3:**
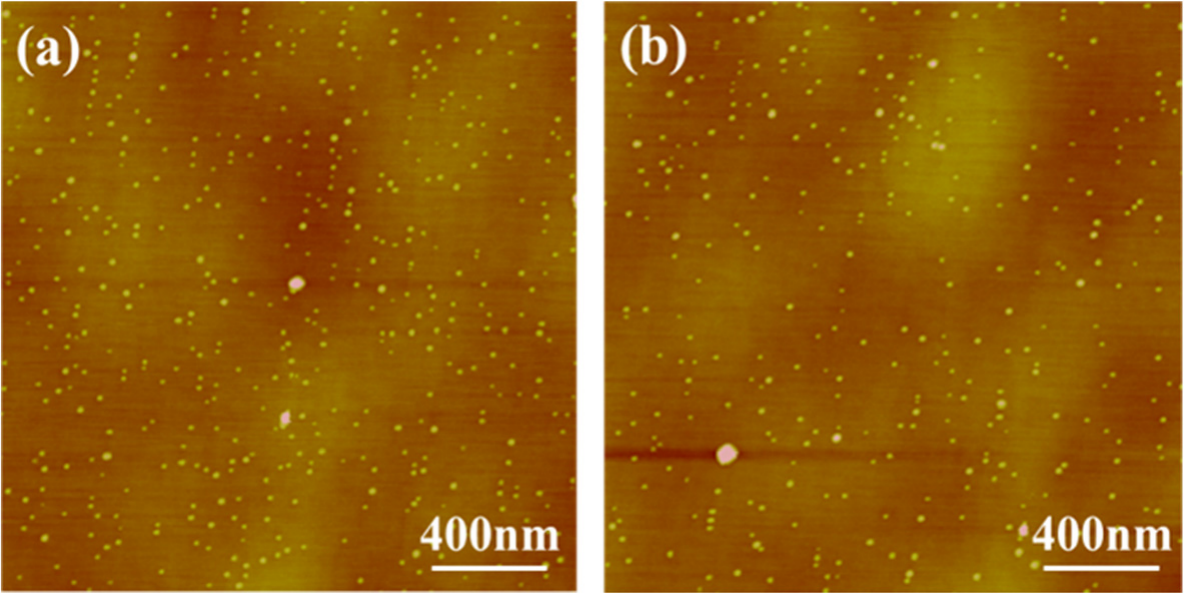
**The 2 × 2 μm AFM images of the fifth layer InAs QDs. (a)** Sample A1 with Ga_0.90_In_0.10_As SRL, **(b)** sample A2 without Ga_0.90_In_0.10_As SRL.

**Figure 4:**
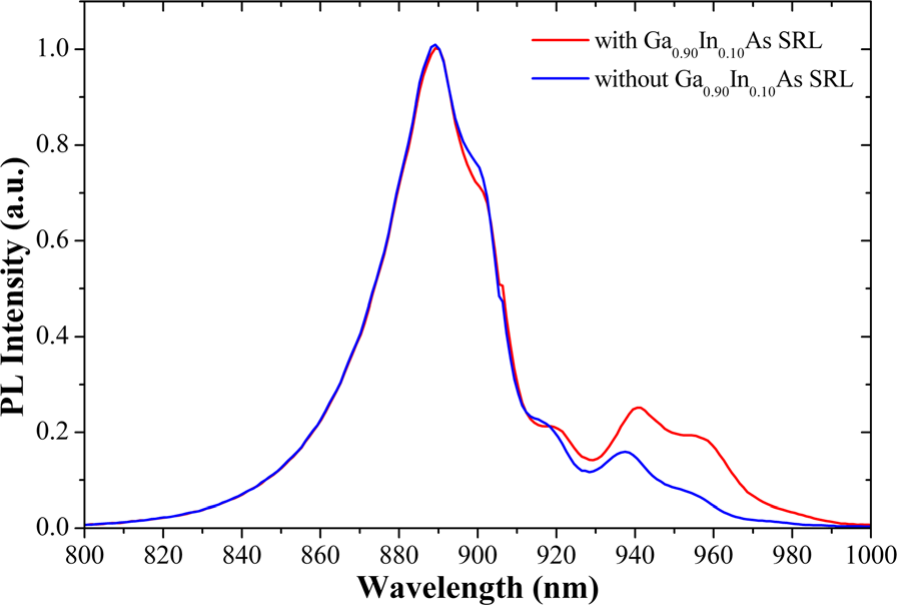
**PL spectra of InAs/GaAs QDs with and without Ga**_**0.90**_ **In**_**0.10**_ **As SRL.**

Figures 3a and b show the 2 × 2 μm AFM images of QDs with and without Ga_0.90_In_0.10_As SRL. The corresponding room temperature PL spectra are plotted in Figure 4. By inserting an Ga_0.90_In_0.10_As SRL, the InAs QD density of sample A1 is increased from 8.1 × 10^9^ cm^−2^ (sample A2) to 1.2 × 10^10^ cm^−2^ (sample A1), which is consistent with the PL intensity. As is well known, the strain fields created by the QDs in the first layer strongly influence the QD nucleation on the second layer [19]. The increasing of the QD density of sample A1 with Ga_0.90_In_0.10_As SRL can be ascribed to the reduction of the accumulated strain field, which is assumed by the strain as interpreted by the XRD results. Correspondingly, the PL intensity of QDs is improved by the inserting of Ga_0.90_In_0.10_As SRL. For one reason, the QD density is increased. Secondly, less defects may be existed in sample A1 due to the decrease of lattice mismatch, leading to less SRH recombination centers [20]. These two reasons induced a better optical performance of sample A1. In addition, the PL peak of sample A1 exhibits a red shift of about 3 meV with respect to samples A2. The following reasons may need to be considered: Firstly, the relief of the compressive strain conducted a 1.94 meV red shift to the band energy, which is calculated by the following equation [21],

3 $$ \varDelta {E}_0=1.71\varepsilon (eV) $$

Secondly, the Ga_0.90_In_0.10_As SRL could suppress the In/Ga inter-diffusion [22] compared to GaAs, thus conducting a less blue-shift. At last, the less quantum confinement of GaInAs [23] than GaAs will result in a red shift to the QDs.

**Figure 5:**
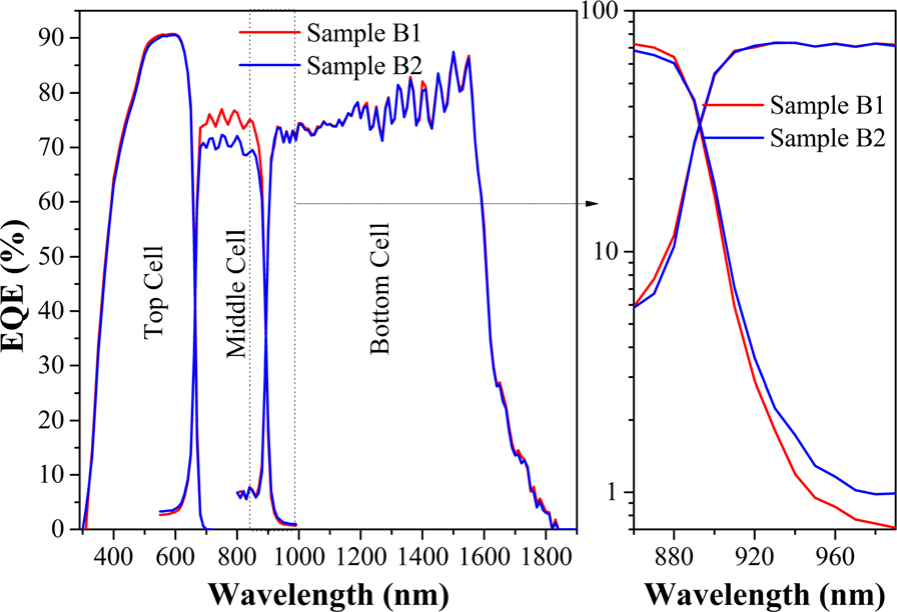
**EQE of QD TJSC with and without Ga**_**0.90**_ **In**_**0.10**_ **As SRL.**

As shown in Figure 5, the top cell in the QD TJSC with and without a Ga_0.90_In_0.10_As SRL yields EQE of 12.65 and 12.55 mA/cm^2^, respectively. The relative lower EQE of the top cell without Ga_0.90_In_0.10_As s SRL may be caused by the deteriorated crystalline quality of GaInP [24], which was affected by the strained InAs/GaAs QD SL in the middle cell. The EQE of the middle cell is 11.16 and 10.51 mA/cm^2^ in sample B1 and sample B2, respectively, and exhibits absorption edge at 950 nm. This extended absorption range is due to the QDs absorption, whose threshold wavelength is in agreement with the QD ground state transition. The spectral response of the wetting layer and QD ground state transitions is approximately 5% and approximately 1%, respectively. Remarkably, the middle cell with Ga_0.90_In_0.10_As SRL yields higher EQE response (about 75%) than that without Ga_0.90_In_0.10_As SRL (about 70%) in the spectral region below 880 nm, which can be attributed to the less defects in sample B1 and thus reduced the SRH recombination of photon generated carriers. The EQE of the bottom cell in the QD TJSC with and without Ga_0.90_In_0.10_As SRL shows an equal value at 17.2 mA/cm^2^ and a spectral response of approximately 73%. In comparison with top cell, the Ge bottom cell has a relative lower spectral response. Because the Ge bottom cell is indirect band gap semiconductor that has a short minority carrier diffusion lengths and short minority carrier lifetimes [25]. In a word, the short-circuit current (I_sc_) of the QD TJSC in this case is limited by the middle cell, suggesting that the effects of Ga_0.90_In_0.10_As SRL on current of the middle cell is significant to the final I_sc_ of the QD TJSC.

**Figure 6:**
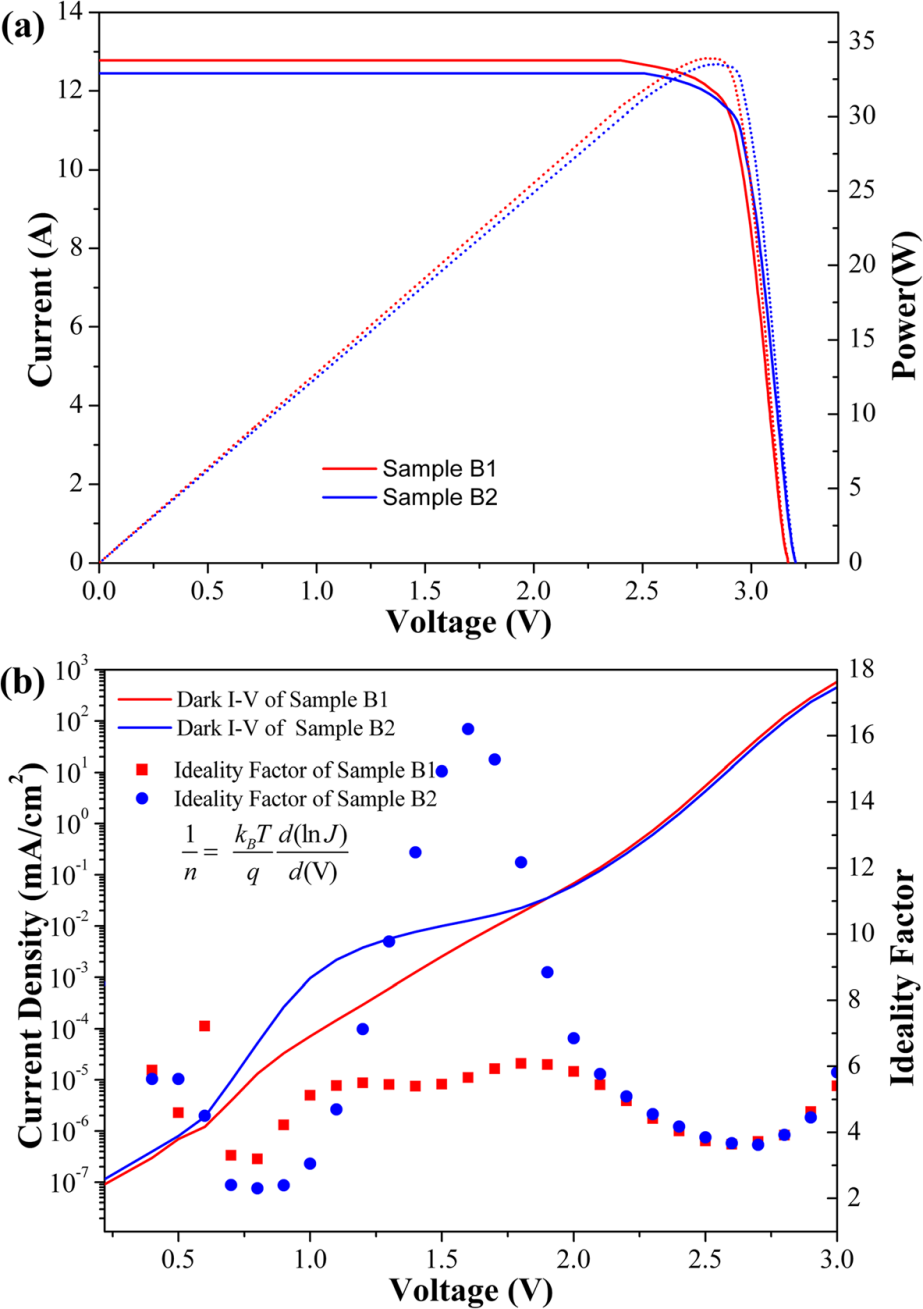
**The I-V measurements and dark I-V curves. (a)** I-V measurements at 1,000 suns AM 1.5D of QD TJSC with and without Ga_0.90_In_0.10_As SRL, **(b)** dark I-V curves and the corresponding ideality factor of QD TJSC with and without Ga_0.90_In_0.10_As SRL.

Figure 6a shows the I-V characteristics of the QD TJSC under 1,000 suns. The values of open-circuit voltage (V_oc_), fill factor (FF), short-circuit current (I_sc_), and convention efficiency (η) are listed in Table 1. The I_sc_ of sample B1 is 12.79 A, and a 2.73% higher than that of sample B2. As mentioned above, the crystalline quality is improved by inserting of a Ga_0.90_In_0.10_As SRL with reduced the defects. So the improvement of I_sc_ is assumed due to the reduction in SRH recombination that originated from the defects, which is in well agreement with the EQE characterization as shown in Figure 5. To further clarify this speculation, the dark I-V was measured by Keithley system and displayed in Figure 6b. As we can see that the two I-V curves are almost overlapped with each other. The only difference is the hump that appears at the sample B2 with respecting the different epitaxial structure of Ga_0.90_In_0.10_As SRL layer. Know to all, the corresponding ideality factor (n) as a function of applied voltage is described as Equation 4:

**Table 1:** **The open-circuit voltage (V**_**oc**_ **), fill factor (FF), short-circuit current (I**_**sc**_ **), and conversion efficiency (η) of QD TJSC**

**No.**	**I**_**sc**_ **(A)**	**V**_**oc**_ **(V)**	**FF(%)**	**η(%)**
Sample B1	12.79	3.17	83.69	33.91
Sample B2	12.45	3.20	84.01	33.51

4 $$ \frac{1}{n}=\frac{k_BT}{q}\frac{d(lnJ)}{d(V)} $$

Where the k_B_ is the Boltzmann constant, q is the elementary charge, and J is the current density. As illustrated in Figure 6b, the ideality factor of the sample without Ga_0.90_In_0.10_As SRL is varied from 2 to 16 at a low forward voltage bias (1.2~1.9 V hump area), but the one from sample B1 almost remains constant. The large ideality factor (*n* > 10) in the sample without Ga_0.90_In_0.10_As SRL suggests that more SRH recombination is occurred [26]. Notably, the V_oc_ of sample B1 is 30 mV lower than that of sample B2. The difference in V_oc_ between samples with (B1) and without (B2) Ga_0.90_In_0.10_As SRL is larger than that in InAs ground state energy observed in PL. So the fact that the In_0.1_Ga_0.9_As SRL has lower band energy relative to GaAs and consequently decreases the thermal activation energy of confined carriers need to be considered in the case. Nevertheless, by inserting Ga_0.90_In_0.10_As SRL, conversion efficiency of GaInP/Ga(In)As/Ge TJSC embedded with InAs QDs is improved by 1.19% relatively and arrive at 33.91% under 1,000 suns AM 1.5D.

## Conclusions

In summary, the InAs/GaAs QDs structures embedded in GaInP/Ga(In)As/Ge TJSC with and without Ga_0.90_In_0.10_As SRL were investigated. The QD density was increased and a red shift of the QD illumination peak was observed by inserting a Ga_0.90_In_0.10_As SRL. Also, the strain was reduced by the inserting of Ga_0.90_In_0.10_As SRL. Although a reduction in V_oc_ was observed in the sample B1 due to the decrease of thermal activation energy of confined carriers of Ga_0.90_In_0.10_As SRL and a reduction in the total energy band gap, the conversion efficiency of QD TJSC is improved by 1.19% relatively via inserting the Ga_0.90_In_0.10_As SRL. The cell with Ga_0.90_In_0.10_As SRL showed an improved device quality and reduced SRH recombination of photon generated carriers, thus resulted in a large increase of I_sc_. Finally, the QD TJSC with Ga_0.90_In_0.10_As SRL has demonstrated a conversion efficiency of 33.91% at 1,000 suns AM 1.5D.

## Abbreviations

AFM: atomic force microscope
ARC: anti-reflection coating
I-V: current-voltage
EQE: external quantum efficiency
η: efficiency
FF: fill factor
HRXRD: high resolution x-ray diffraction
MOCVD: metal organic chemical vapor deposition
QDs: quantum dots
RT: room temperature
I_sc_: short-circuit current
SRH: Shockley-Read-Hall
SRL: strain reducing layer
SL: super lattice
TJ: tunnel junction
TJSC: triple junction solar cell
V_oc_: open-circuit voltage
